# A Numerical Wear Simulation Method of Reciprocating Seals with a Textured Rod

**DOI:** 10.3390/ma13194458

**Published:** 2020-10-08

**Authors:** Hongliang Ran, Di Liu, Shaoping Wang

**Affiliations:** 1 School of Automation Science and Electrical Engineering, Beihang University, Beijing 100083, China; leorhl@163.com (H.R.); liudi54834@buaa.edu.cn (D.L.); 2 Equipment Support Department, Logistics University of PAP, Tianjin 300309, China; 3 Beijing Advanced Innovation Center for Big Data-based Precision Medicine, Beihang University, Beijing 100191, China; 4 Ningbo Institute of Technology, Beihang University, Ningbo 315800, China

**Keywords:** reciprocating seal, texturing rod, seal wear, lubrication characteristics, leakage

## Abstract

Reciprocating rod seals are widely used in the hydraulic actuator to prevent the leakage of fluid. The sealing lip profile changes with the seal wear, resulting in an increase in the leakage. A texturing rod changes the lubrication characteristics of the seal, so it affects the wear and leakage of the seal. A numerical simulation method is proposed to investigate the wear of the hydraulic reciprocating seal with textured rods. Several kinds of macro-cavity textures on the rod surface, including circle, square and triangle shapes, have been simulated and discussed. The effects of three shape parameters including area ratio, depth, and ratio of the axial length to the circumferential length on the seal wear are analyzed in detail. The texturing rod slightly increases the seal wear, but decreases the seal leakage. When the rod speed is increasing, the wear time rates of the seal increase, while the wear distance rates decrease, regardless of the texture shapes. When the texture area ratio is increasing, the wear of the reciprocating seal increases. Seal wear decreases with an increasing texture depth during the outstroke, however, it increases during the instroke. The ratio of the axial length of the macro-cavity to the circumferential length has no effect on the seal wear.

## 1. Introduction

Elastic seals are critical components and widely used in hydraulic systems to prevent the leakage of the fluid [1,2]. As one of the dynamic elastic seals, reciprocating rod seals are widely used in hydraulic actuators [3,4]. If the seal fails, the leakage of actuator would not only pollute the environment, but also cause the loss of working capacity. Thus, the hydraulic system is significantly affected by the performance of the reciprocating seal.

Many published research about the reciprocating seals, such as the O-ring [5], VL seal [6,7], and so on [8,9], have clarified the relationships between the performance with the operating conditions. With improvements in the laser surface processing technology, laser texturing surface for improving the performances of mechanical components has attracted extensive attention [10,11,12]. Especially, improving seal performances by a texturing rod is an important topic for the seals. Huang et al. [13] analyzed the performances of the reciprocating seal with plunge ground rods. The contact pressure, friction force and fluid transport are simulated and discussed by a mixed lubricating model. The seal performances of the reciprocating seal with textured rod have also been numerically studied in Ref. [14]. Gadari et al. [15] numerically analyzed the sealing mechanism of reciprocating seal with grooved rod. The lubrication film and fluid pressure distributions are simulated by the modified Reynolds equation considering the cavitation on the sealing zone. A comparison between experimental and numerical results has shown that the method is more accurate than the inverse hydrodynamic lubrication (IHL) model. In addition, Guo et al. [16,17] investigated the effects of the textured shafts on the performances of rotary lip seal. It is found that the textured shafts would change the pumping rate and friction torque of the shaft lip seal.

Since the seal is operating under mixed lubricating conditions, the wear of the reciprocating seal is unavoidable due to friction and wear, the sealing lip is thus continuously changed when the seal is working [18]. With the change of the lip profile, the contact width and contact pressure on the sealing zone are changed accordingly. Since the sealing performance is significantly dependent on the contact width and contact pressure, the wear of the sealing lip would lead to a continuous change of the sealing behaviors, including friction, leakage, and so on [19].

Some previous research on the seal wear mainly concentrate on the experimental aspects. Combing the experiment with the numerical model, the influence of the wear of the seal lip on the performance of the shaft lip seal was studied [18]. The wear properties of the seal materials were investigated by experiments, including accelerated wear [20], and two-body abrasive wear [21], and so on [22,23]. However, since there are many parameters to be considered and controlled in the experiments, the experimental studies are normally time consuming and expensive. Numerical approaches have thus been used to study on the wear of the seal. Due to the widely used of Finite element method (FEM) in the structural analysis [24], a combination of the FEM and Archard wear model is normally adopted to simulate the seal wear [25]. The contact pressure is calculated by finite element analysis (FEA). The wear depth of the seal lip is solved by the Archard wear model. Békési et al. [26,27] simulated the wear process of the reciprocating seal. Considering the effects of temperature, a structural and thermal coupling simulation model is developed based on the FEM to study the wear of the seals, including the O-shape seal and rectangular-section seal [28,29]. The continuous wear process is approximated as a discrete set of time. The wear depth is calculated by the Archard wear model and used to update the seal lip geometry. Frölich et al. [23] presented a macroscopic simulation model for analyzing the contact behavior of a rotary lip seal, considering the interaction of temperature, friction and wear.

However, the method of combing the FEM and Archard wear model neglects the lubricating effects on the wear of the seal. In fact, lubrication characters and wear of the seal are strongly coupled. To this end, the effects of the lubrication on the seal wear should not be neglected when analyzing the seal wear. To investigate seal wear under different lubricating conditions, a numerical wear model is proposed in Ref. [30] based on the elasto-hydrodynamic (EHD) lubrication model, Archard wear model and macro contact model. The relationship between the lubricating characteristics and the seal wear was analyzed. In addition, a multiscale simulation model was made to analyze the relationship between seal wear and lubrication characteristics in Refs. [31,32]. In the simulations, macro contact load is analyzed by the macroscale finite element model. Asperity contact and hydrodynamic pressures are calculated by a mixed thermal EHD lubrication model. The Archard model is modified to calculate the seal wear.

For the textured surface, Xiong et al. [33] studied the sliding wear of polytetrafluoroethylene (PTFE) experimentally. Qi et al. [34] investigated the effects of the textured steel surface on the tribological properties of PTFE composite in dry friction. Moreover, the effects of the textured shafts on the wear of the rotary lips seal were analyzed in Ref. [35]. However, it is difficult to find a study on the effects of the textured rod on the wear of the reciprocating seal. In addition, since the experimental studies are always time consuming and labor consuming, a numerical wear simulation method is presented in this paper for reciprocating seals with textured rods. Three common kinds of texture shapes, such as circle, square, and triangle, are modeled and investigated. The effects of the lubricating characteristic of the reciprocating seal are considered in the numerical model. Under different rod speeds and different texture parameters, the wear of the reciprocating seal is simulated and discussed.

The structure of the rest of the paper is as follows. Rod textures and shape feature parameters are introduced in Section 2. In Section 3, the lubrication model is developed with considering the rod textures. In Section 4, the Archard wear model is modified considering the lubrication effects to solve the seal wear. Section 5 presents the simulation procedure of the proposed method. In Section 6, seal wear with different rod textures are simulated, the effects of rod texture on lubrication characteristics and seal wear are discussed. The effects of rod speed on the seal wear are also investigated. The conclusions are presented in Section 7.

## 2. Rod Surface Texture

**Figure 1:**
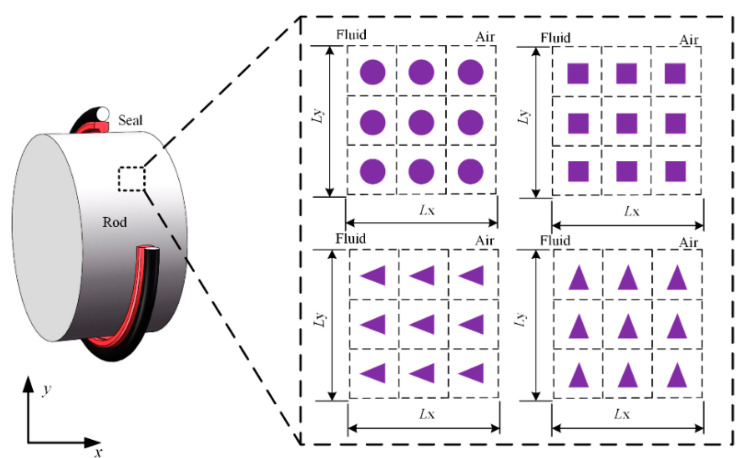
Textured rod surfaces.

As shown in Figure 1, four kinds of micro-cavity textures are discussed in the presented research, including circle, square and triangle shapes. *L*_y_ and *L*_x_ are the lengths of the simulation space in circumferential direction and axial direction. Normally, the axial length of sealing zone of the reciprocating step seal used in this paper is about 0.4–0.8 mm. The smaller the texture is, the more difficult it is for surface manufacturing by the laser texturing techniques. Having many rows of texture in the axial direction contact zone is not practical. Therefore, three texture rows are arranged along the contact width, as shown in Figure 1, the texture dimension is approximately within the tens of micron range.

**Figure 2:**
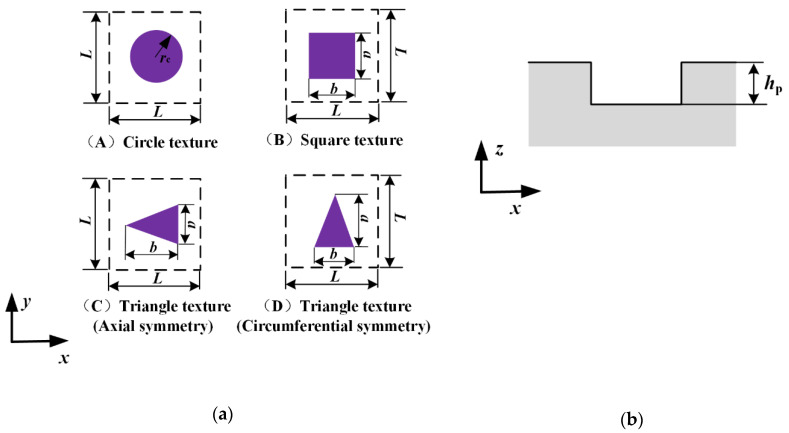
(**a**) Micro-cavity texture geometries and (**b**) cross-section of the texture.

Figure 2 shows the geometries and the cross-section of the textures. The texture area ratio is one of the most important geometric parameters affecting the lubrication characteristics of the seal, which is defined by

(1) $$A=\{\begin{array}{ll} \frac{\pi r_{c}^{2}}{L^{2}}\; & \mathrm{circular}\;\mathrm{texture}\\ \frac{ab}{L^{2}}\; & \mathrm{square}\;\mathrm{texture}\\ \frac{ab}{2L^{2}}\; & \mathrm{triangular}\;\mathrm{texture} \end{array}$$

Furthermore, the shape feature parameter *γ* is defined as the ratio of the axial length of the micro-cavity to the circumferential length, which is given by

(2) $$\gamma =\frac{b}{a}$$

Generally, there would be fluid in the micro-cavity when the rod moving, which would affect the lubrication characteristics of the rod seal. It is assumed that the micro-cavity is full of lubrication fluid during outstroke and instroke. Hence, the surface of the texture rod should be described mathematically to analyze the lubricating characteristics. The rod surface height is given by

(3) $$h_{r}=\{\begin{array}{l} 0\;\mathrm{in}\;\text{non-textured}\;\mathrm{zone}\\ h_{p}\;\mathrm{in}\;\mathrm{textured}\;\mathrm{zone} \end{array}$$

where *h*_p_ is depth of the micro-cavity.

## 3. Lubrication Analysis

**Figure 3:**
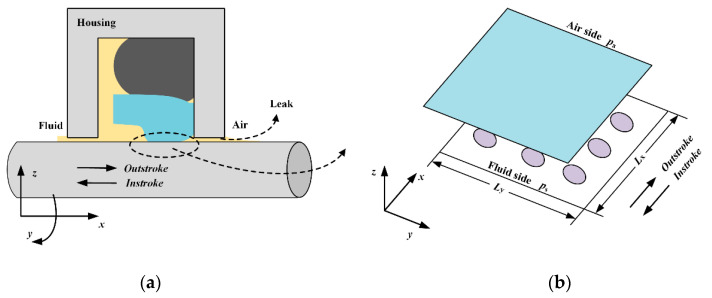
(**a**) Structure of reciprocating seal [31]; (**b**) Cartesian coordinate system on sealing zone.

The lubricating film is so thin compared with the rod radius that the effects of curvature can be neglected. Therefore, a Cartesian coordinate system is applied, as shown in Figure 3. The x-axis, y-axis and z-axis indicate the axial direction, circumferential direction, and radial direction, respectively. After the running-in polished period, the rod’s surface roughness is much smaller compared to the seal surface roughness. So, the surface roughness of the rod is neglected when analyzing the lubricating characteristics of the reciprocating seal.

### 3.1. Analysis of Fluid

When the fluid pressure drops below the cavitation pressure, cavitation will occur on the sealing zone. This phenomenon can be modeled by introducing the cavitation index *F* and universal variable *Φ* in the Reynolds equation [36]. Moreover, considering the effects of the sealing lip roughness, the Reynolds equation should be improved [37]. Therefore, the Reynolds equation used in the presented research is

(4) $$\frac{\partial }{\partial x}\left(\phi_{x}\frac{h^{3}}{\mu }\frac{\partial }{\partial x}(F\Phi )\right)+\frac{\partial }{\partial y}\left(\phi_{y}\frac{h^{3}}{\mu }\frac{\partial }{\partial y}(F\Phi )\right)=6u\frac{\partial }{\partial x}\left\{\left[1+(1-F)\Phi \right]\left(h_{T}+\sigma \phi_{s.c.x}\right)\right\}$$

where *μ* is fluid viscosity, *h* is film thickness, *u* is rod speed and *σ* is seal roughness. *ϕ*_x_ and *ϕ*_y_ are the pressure flow factors and *ϕ*_s.c.x_ is the shear flow factor. When *Φ*<0, means cavitation zone, *F* = 0 and the fluid pressure *p*_f_ = *p*_cav_, and *p*_cav_ is the cavitation pressure which is assumed to be zero in the presented research. When *Φ* > 0, means non-cavitation zone, *F* = 1 and the fluid pressure *p*_f_ = *FΦ*. At the fluid side, *Φ*(0,y) = *p*_s_. At air side, *Φ*(*L*_x_,y) = *p*_a_. *p*_a_ is the ambient pressure and *p*_s_ is the sealed pressure. Furthermore, *Φ*(x,0) = *Φ*(x, *L*_y_).

When the asperity height of the seal surface follows Gaussian distribution, the average truncated film thickness *h*_T_ is given by:

(5) $$h_{T}=\frac{h}{2}+\frac{h}{2}erf(\frac{h}{\sqrt{2}})+\frac{1}{\sqrt{2\pi }}e^{-h^{2}/2}$$

The piezo–viscous properties of the film is described by Roelands equation [4], as

(6) $$\mu =\mu_{0}\exp\left\{\left(\ln\mu_{0}+9.67\right)\left[{\left(1+\frac{p_{f}}{p_{z}}\right)}^{\alpha }-1\right]\right\}$$

where *p*_z_ and *α* are constants and *μ*_0_ is the fluid viscosity under the ambient pressure. Furthermore, *p*_z_ = 0.196 GPa and *α* = 0.5–0.98 in the presented research.

The film thickness is given by

(7) $$h(x,y)=h_{0}(x,y)+h_{r}(x,y)+\Delta h(x,y)$$

where *h*_0_ is the initial film thickness, which can be obtained by inversing the asperity contact model through the look-up table method [7]. Δ*h* is the micro deformation of the sealing lip surface.

The fluid flow rate is given by

(8) $$q=\frac{\pi D_{\mathrm{rod}}}{12L_{y}}\int_{0}^{L_{y}}\left\{-\phi_{x}\frac{h^{3}}{\mu }\frac{dp_{f}}{dx}+6u\left[1+(1-F)\Phi \right]\left(h_{T}+\sigma \phi_{s.c.x}\right)\right\}dy$$

where *D*_rod_ is rod diameter.

### 3.2. Analysis of Asperity Contact

Greenwood–Williamson (G–W) contact model is adopted to calculate the asperity contact pressure, which is given by [38],

(9) $$p_{c}=\frac{4}{3}ER^{1/2}\eta \int_{h}^{\infty }f(y){\left(y-h\right)}^{3/2}dy$$

where *p*_c_ is asperity contact pressure, *R* and *η* are the asperity radius and the asperity density of the seal, respectively. The equivalent Young’s modulus *E* is given by

(10) $$E=\frac{E_{seal}}{1-\upsilon_{seal}^{2}}$$

where *υ*_seal_ is Poisson’s ratio of the seal and *E*_seal_ is Young’s modulus of the seal.

It is assumed that the seal surface roughness follows Gaussian distribution, and the probability density function is given by

(11) $$f(y)=\frac{1}{\sqrt{2\pi }\sigma_{s}}\exp\left(-\frac{y^{2}}{2\sigma_{s}^{2}}\right)$$

where *σ*_s_ is the equivalent standard deviation of surface roughness, which is given by [39],

(12) $$\sigma_{s}^{2}=\sigma^{2}-\frac{3.3717\times 10^{-4}}{R^{2}\eta^{2}}$$

### 3.3. Analysis of Deformation

**Figure 4:**
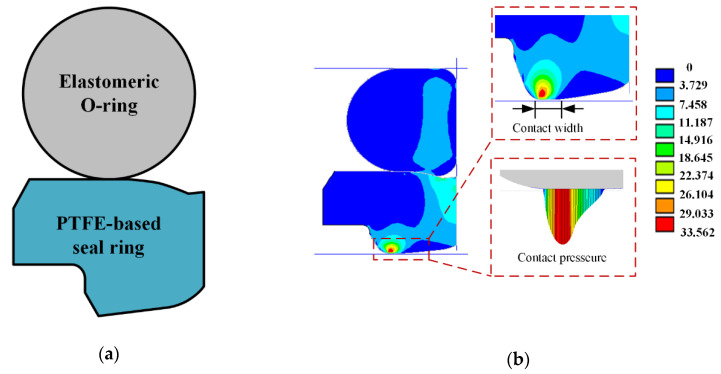
(**a**) A step seal; (**b**) finite element analysis (FEA) model of the rod seal.

Macroscopic deformation on the seal lip surface occurs due to the interference fitting and the sealed pressure. The analytical solution of the macroscopic deformation is too difficult to be obtained, FEA is thus adopted to simulate the macroscopic deformation. In the presented research, a typical step rod seal is studied, Figure 4a. The O-ring is made of nitrile rubber, which is typical hyper elastic and nonlinear rubber material. The Mooney–Rivlin hyper elastic model with two parameters is used to describe the relationship between the stress and strain of the O-ring [40],

(13) $$\tilde{W}=C_{10}(I_{1}-3)+C_{01}(I_{2}-3)+(J-1)/d$$

where $\tilde{W}$ is strain energy density. *I*_1_ is the first deviatoric strain invariant and *I*_2_ is the second deviatoric strain invariant. *J* is relating to the elastic deformation gradient. *C*_10_, *C*_01_ and *d* are Mooney–Rivlin coefficients.

The seal ring contacts with the rod is made of PTFE-based material. Compared with the nitrile rubber, the Young’s Modulus of PTFE is much bigger. Thus, the linear elastic model is used in the finite element analysis of the PTFE-based ring. Housing and rod are made of steel, Young’s Modulus is hundreds of GPa. Comparing with step seal, Young’s Modulus is so high that it is assumed the housing and rod are rigid in the FEA.

Figure 4b shows a two-dimensional-axisymmetric finite element model of the step seal. By the affecting of the radial interference fitting and sealed pressure, the seal is deformed and the sealing lip is pressed on the rod’s surface. In this research, the material parameters of the seal are as shown in Table 1. Finite element analysis software ANSYS was used to analyze the macroscopic deformation of the seal. By the analysis of finite element, the static contact pressure and contact width on the sealing zone are obtained. Figure 4b shows the simulating results with 5 MPa sealed pressure.

Under the action of the interference fitting and sealed pressure load, the seal is deformed, as shown in Figure 4, which we call macroscopic deformation. When the rod is moving, microscopic deformation on the sealing lip will occur duo to the hydrodynamic effects of the lubricating film on the sealing zone. Generally, the lubricating film on the sealing zone is only a few microns. So, the microscopic deformation of the sealing lip caused by the lubricating film is very small. Compared with the macro deformation, the micro deformation on the sealing lip so small that it is assume the micro deformation has no effects on the macroscopic deformation of the seal. Namely, the static contact pressure and contact width will not change with the microscopic deformation of the sealing lip.

The simulation of the microscopic deformation is divided into two parts. Firstly, the calculation of the microscopic deformation on seal surface with a smooth rod is produced. Since the reciprocating seal is under the mixed operating conditions, asperity contact occurs and the ratio of the film thickness to the surface roughness is less than three. Therefore, the micro deformation of the seal lip caused by the film is very little. Compared with the micro deformation of the seal lip, the total size of the seal is so large that the seal can be considered as a semi-infinite body [4]. Therefore, the calculation of the seal microscopic deformation is given by

(14) $$\Delta h(x,y)=\iint_{\begin{array}{c} \mathrm{Simulation}\\ \mathrm{space} \end{array}}\left(\frac{1-\upsilon_{seal}^{2}}{\pi E_{seal}}\frac{\Delta p(\zeta ,\vartheta )}{\sqrt{{\left(x-\zeta \right)}^{2}+{\left(y-\vartheta \right)}^{2}}}\right)d\zeta d\vartheta$$

The local pressure difference Δ*p*(x, y) is given by

(15) $$\Delta p(x,y)=p_{f}(x,y)+p_{c}(x,y)-p(x,y)$$

where *p* is the static contact pressure.

Secondly, the microscopic deformation of the seal caused by the textures on the rod is simulated. After the calculation in the first simulation, the pressure difference in the non-textured zone is zero, the sum of the pressure differences in the textured zone is

(16) $$F_{sum}=\iint_{textured\;zone}\left(p_{f}(x,y)+p_{c}(x,y)\right)-p(x,y)dxdy$$

Since the seal is viscoelastic, when the rod moving there is not enough time for the seal surface to fall into the micro-cavity of the textured rod. Hence, the pressure difference in the textured zone is undertaken by the non-textured zone [14]. During the movement of the rod, the texture on the sealing zone is changed all the time. It is assumed that the force difference on the sealing zone is spread equally across the simulation area, the deformation of the seal lip surface is then calculated by Equation (14) with the following pressure difference

(17) $$\Delta p(x,y)=\frac{F_{sum}}{L_{x}L_{y}}$$

## 4. Wear Analysis

The Archard model is commonly applied to calculate the seal wear [23,24,25,26,27,28,29,30]. In the Archard wear model, the wear volume is calculated by

(18) $$V=\frac{K}{H}WS$$

where *V* is the material wear volume, *K* is the wear coefficient, *S* is the relative sliding distance of surface, *W* is the normal load on the surface, *H* is the hardness of softer material.

Define wear modulus as

(19) k=K/H

Wear equation can be rewritten as

(20) V=kWS

Wear depth is given by

(21) $$h_{W}=kp_{n}S$$

where *p*_n_ is the normal contact pressure.

**Figure 5:**
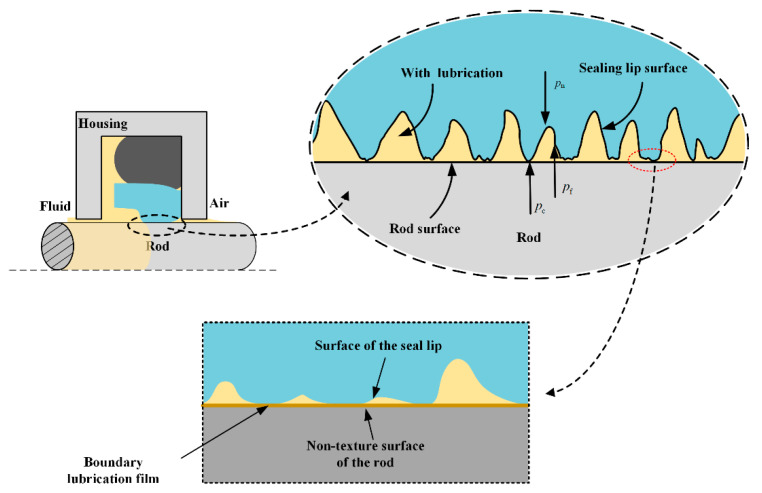
Detailed lubrication status on the sealing zone.

Generally, the wear modulus is obtained experimentally under a specific condition, in the wear analysis, it is assumed to be constant. However, as shown in Figure 5, the reciprocating rod seal is normally operated in mixed lubrication conditions. With the change of the operating conditions, the lubrication characteristics of the seal on the sealing zone are changed accordingly. Especially, when the rod is textured, the lubrication characteristics change significantly. Therefore, the previous method is not suitable for analyzing the reciprocating seal with the textured rod, the Archard model should be modified for considering the lubrication.

In the mixed lubrication conditions, the normal load of the sealing lip is composed of the fluid and asperity contact pressures, namely,

(22) $$p_{n}=p_{f}+p_{c}$$

When the fluid is so clean that there is no particle in the lubricating film, the seal wear caused by the fluid pressure can be considered to be zero [35]. In this case, only asperity contact would cause seal wear. The wear depth of the reciprocating rod seal is calculated by

(23) $$h_{W}=kp_{c}S$$

where *p*_c_ is predicted by the lubrication analysis.

The contact of the seal with the rod surface at the asperity point is not direct and dry. Since there is lubrication fluid existing in the sealing region, there is a boundary lubrication film at the contact point, as shown in Figure 5. Therefore, the wear modulus *k* of the sealing lip should be obtained by the boundary lubrication experiment of the PTFE-based material sliding on a smooth face. According to the Ref. [41,42], the value of the wear modulus of the PTFE-based material is approximately 10^−4^–10^−6^ mm^3^/Nm. In the presented research, the wear modulus of the PTFE-based ring is assumed to be 1.2 × 10^−5^ mm^3^/Nm.

When the rod is moving, the lubricating characteristic of the sealing zone changes with time because the rod surface is textured. The asperity contact pressure of the seal changes all the time with the rod moving. So, the average asperity contact pressure of a certain stroke is adopted to calculate the seal wear. Here, the stroke can be assumed to be equal to the texture length *L*, and the continuous motion of the piston rod is divided into *n* states. The wear depth of the seal is given by

(24) $$h_{W}=k{\bar{p}}_{c}L$$

where

(25) $${\bar{p}}_{c}=\frac{1}{n}\sum_{i=1}^{n}{\left(p_{c}\right)}_{i}$$

The wear volume is given by

(26) $$V=\frac{\pi D_{\mathrm{rod}}}{L_{y}}\iint h_{W}dxdy$$

In the presented research, the wear time rate is given by

(27) $$r_{t}=\frac{dV}{dt}$$

The wear distance rate is given by

(28) $$r_{s}=\frac{dV}{dL}$$

## 5. Procedure of the Simulation Method

**Figure 6:**
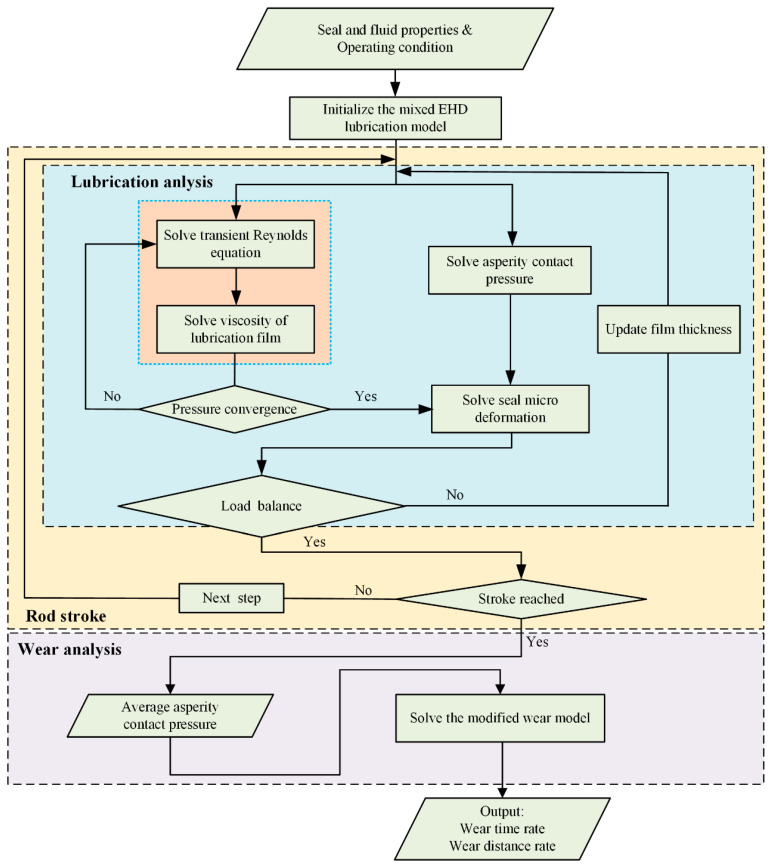
Scheme of computational procedure.

The computational procedure is presented in Figure 6. The mixed lubricating model is firstly solved, then the wear model is solved based on the lubrication analysis. In the lubrication analysis, the fluid mechanics, asperity contact mechanics, and the micro deformation of the sealing lip are strongly coupled. In order to solve this coupling problem, an iterative method is adopted in the numerical analysis.

At the beginning of the numerical analysis, static contact pressure and contact width are obtained by FEA with the software ANSYS. Initial film thickness *h*_0_ is obtained by inversing Equation (9). In the solving of Equation (9), asperity contact pressure is assumed to be equal to the static contact pressure. There are three loops in the analysis of the lubrication of the reciprocating seal. The innermost loop is used to solve the fluid pressure of the film, including the Reynolds equation and Roelands equation. When solving the Reynolds equation, a finite volume method is applied to discretize Equation (4), and the tri-diagonal matrix algorithm (TDMA) method is applied to solve the finite volume discrete equations. More details can be found in Ref. [43]. In the middle loop, contact pressure is solve by Equation (9), and micro deformation on the seal lip is solved by Equations (14)–(17). Then, the follow convergence criterion equation is solved,

(29) $$\frac{\left|\iint p(x,y)dxdy-\iint \left(p_{f}(x,y)+p_{c}(x,y)\right)dxdy\right|}{\iint p(x,y)dxdy}\leq \varepsilon$$

where *ε* is the convergence tolerance. If the convergence criterion is met, solve the outermost loop, else, update the film thickness and return to the fluid pressure calculation. In the outermost loop, the continuous stroke of the seal is approximated as a discrete set of time. For each time step, the asperity contact pressure is solved. When the stroke, here assumed to be equal to the texture length *L*, is reached, the average asperity contact pressure is solved to predict the seal wear.

## 6. Results and Discussion

**Figure 7:**
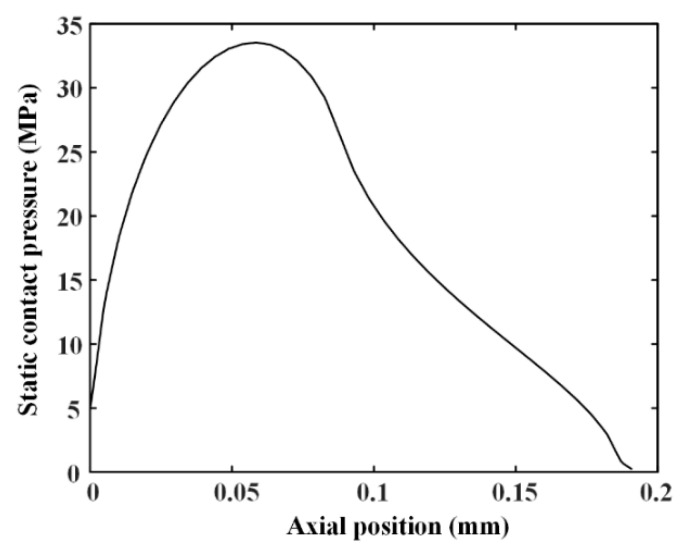
Static contact pressure for 5 MPa sealed pressure.

Wear of reciprocating seals with textured rod can be predicted by the proposed method. The main parameters are shown in Table 2, including seal face roughness, diameter of the rod, and so on. The sealed pressure is 5 MPa. By the FEA, the static contact pressure distributions are obtained, as shown in Figure 7. The maximum of the contact pressure is about 33.562 MPa and the contact width of the seal is about 0.19 mm. Table 3 shows the main geometrical parameters of the rod textures in the simulations.

### 6.1. Validation

**Figure 8:**
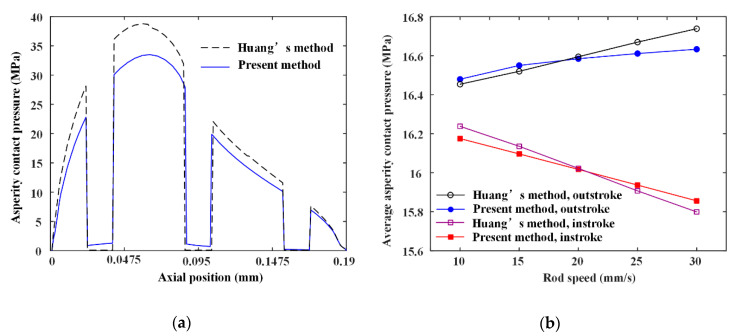
(**a**) Asperity pressure distributions comparison; (**b**) average asperity pressure comparison.

In this section, a comparative study of the simulation results between the present model and the model proposed by Huang et al. [14] to verify the validation of the present numerical model and analytical method. Since the seal wear is calculated by the asperity contact pressure, the comparison of the asperity contact pressure between the two methods is carried out. Figure 8a shows the asperity contact pressure distribution on the sealing zone. It can be seen that the result of Huang’s model is bigger than the result calculated by the present model. This is because Huang et al. used a one-dimensional computational model. The pressure differences in the textured zone are only balanced by the asperity contact pressure and fluid pressure in the non-textured zone along the axial direction. In the present research, a two-dimensional computational model is used. The pressure differences in the textured zone is balanced by the asperity contact pressure and fluid pressure in the non-textured zone of the two-dimensional simulation space. However, the lubrication characteristics on the two-dimensional simulation space can be analyzed by reusing the Huang’s model along the y-axis direction. A comparison of the average asperity contact pressure between the present model and Huang’s model is presented in Figure 8b. It shows that the result calculated by the present model agrees well with that calculated by Huang’s model. When the rod’s speed is increasing from 10 mm/s to 30 mm/s, the maximum difference of the average asperity contact pressure between the present model and Huang’s model is about 0.84% during the outstroke, and 0.31% during the instroke.

### 6.2. Effects of Rod Texture on Lubrication

**Figure 9:**
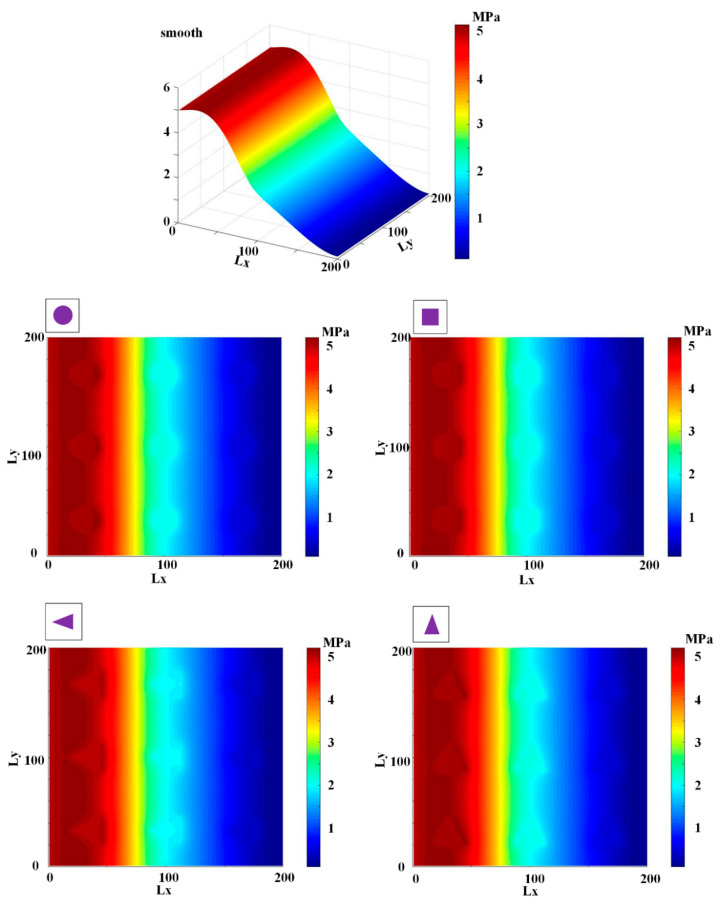
Fluid pressure during outstroke.

**Figure 10:**
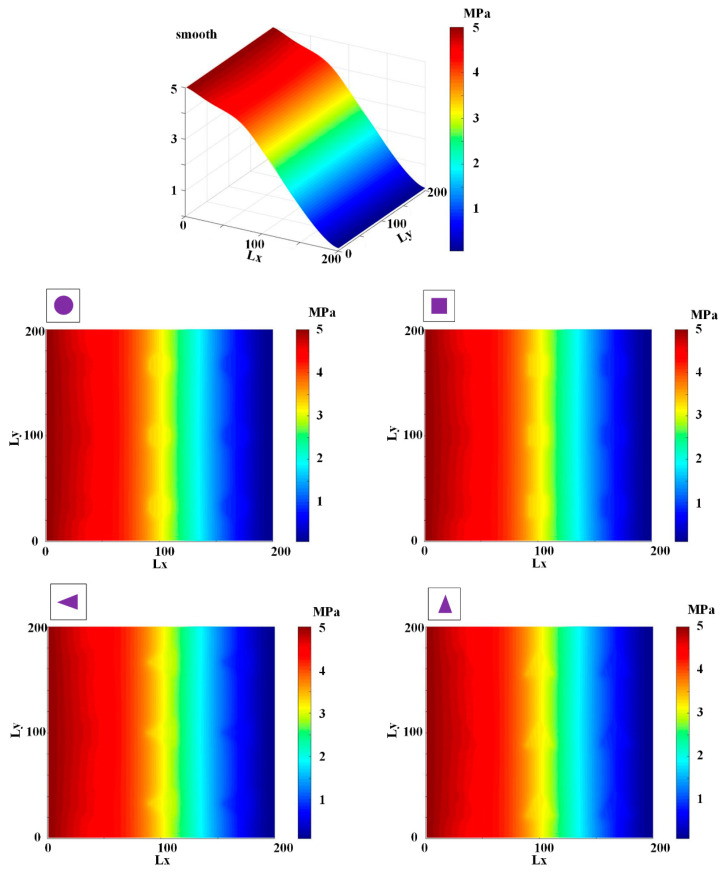
Fluid pressure during instroke.

**Figure 11:**
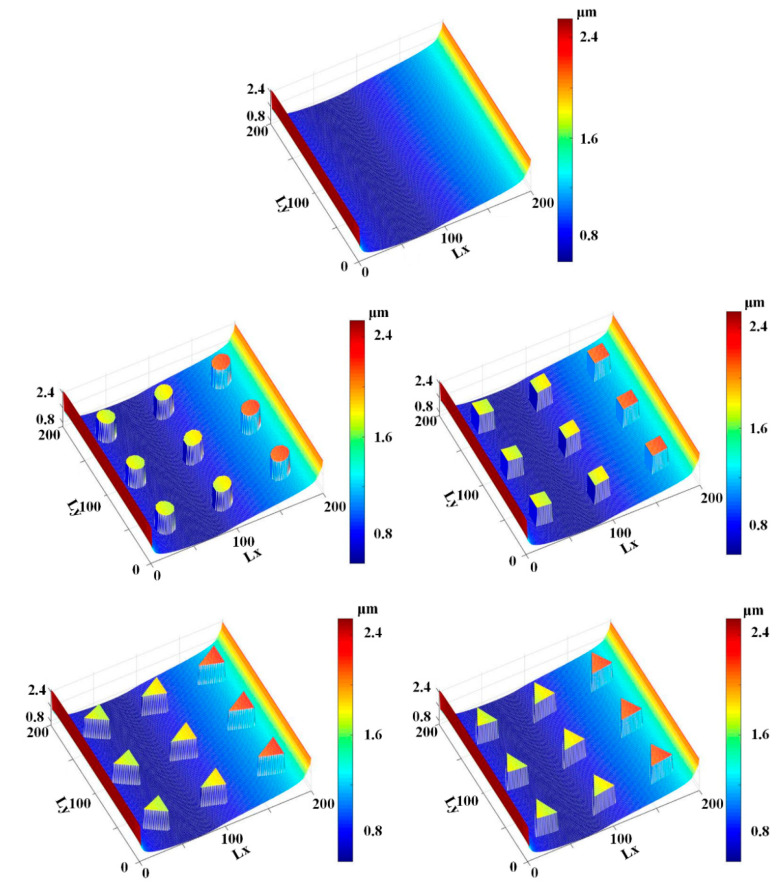
Film thickness during the outstroke.

**Figure 12:**
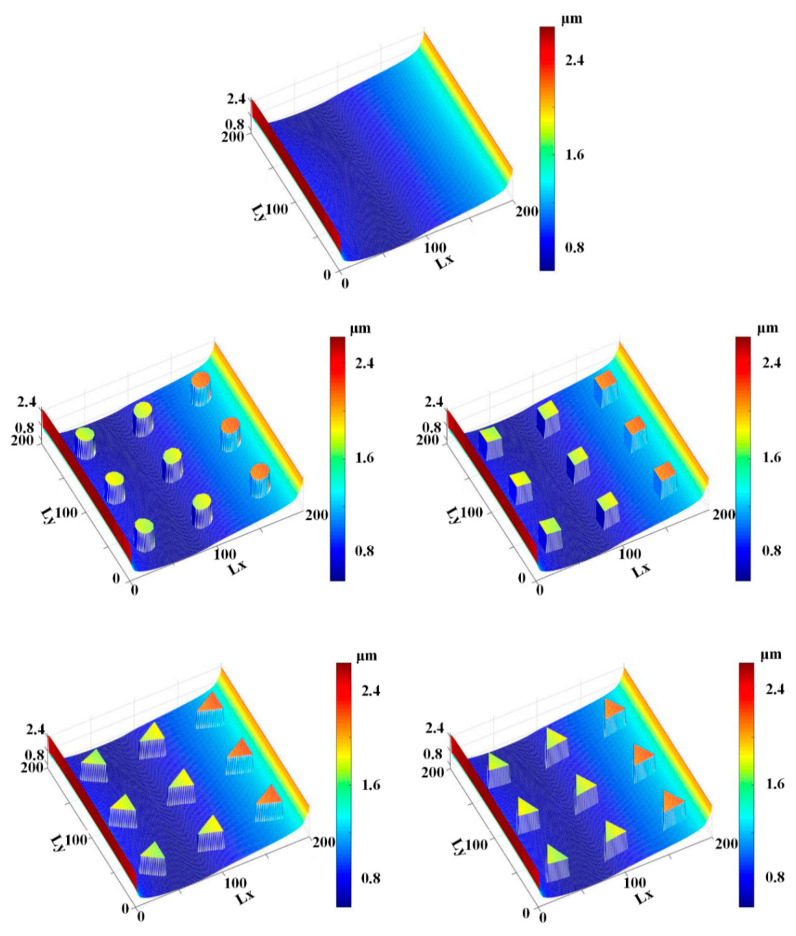
Film thickness during the instroke.

**Figure 13:**
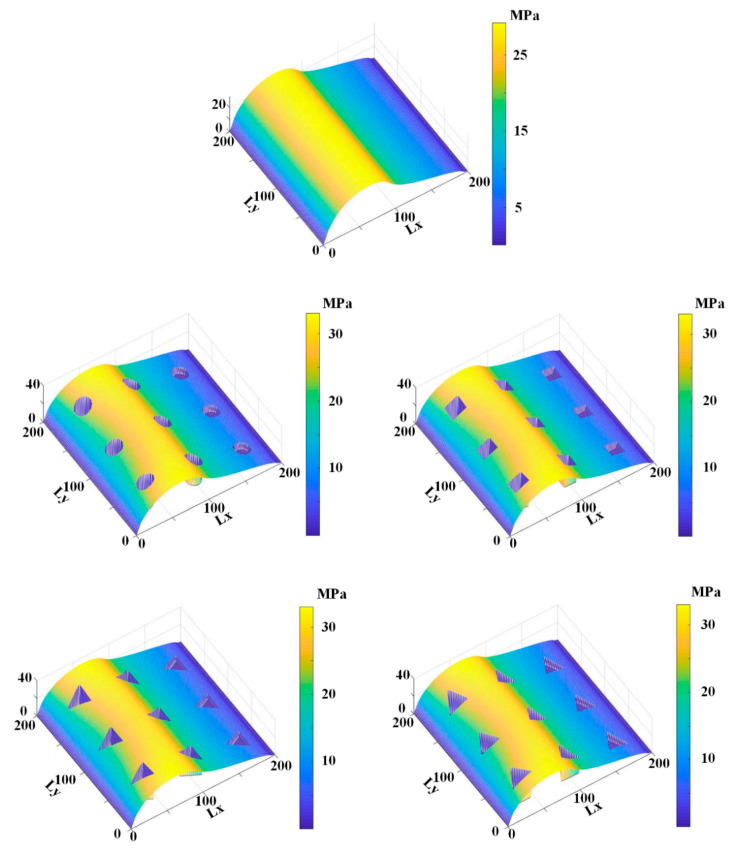
Asperity contact pressure during the outstroke.

Figure 9, Figure 10, Figure 11, Figure 12 and Figure 13 show the fluid pressure distributions, film distributions, and asperity contact pressure distributions for different kinds of textures, respectively. The lubricating characteristics of the reciprocating seal with textured rod are investigated and discussed.

The fluid pressure distributions for different textures rod during outstroke are shown in Figure 9. Note that the fluid pressures of both the smooth and textured rod firstly increase slightly, and then decrease rapidly along the axial direction. During the outstroke, the rod’s speed enhances the hydrodynamic effects and the maximum fluid pressure is thus higher than the sealed pressure. Moreover, the rod texture is so small that the texture has little effect on the fluid pressure. Hence, the fluid pressure distribution of the texture rod, except in the textured zone, is similar to that of the smooth rod. The effects of the textures on the hydraulic pressure is obvious during the outstroke. The fluid pressure of the textured zone near the fluid side is lower than that of the non-textured zone, while near the air side the fluid pressure of the textured zone is higher than that of the non-textured zone, regardless of the texture shapes. So, the texture decreases the hydrodynamic effect near the fluid side, and increases the hydrodynamic effect near the air side.

The fluid pressure distributions for the sealing zone for smooth and textured rods during instroke are illustrated in Figure 10. The results show that the fluid pressure decreases from the sealed pressure to the air pressure along the axial direction. The effects of the textures on the hydraulic pressure is not obvious during the instroke. As the same as the outstroke, the fluid pressure of the textured zone near the fluid side is lower than that of the non-textured zone, while near the air side the fluid pressure of the textured zone is higher than that of the non-textured zone. So, the texture on the rod’s surface decreases the hydrodynamic effect near the fluid side, and increases the hydrodynamic effect near the air side.

Figure 11 and Figure 12 show the simulation results of the lubricating film on the sealing zone with different textured rods, during the outstroke and instroke, respectively. As shown in Figure 11, during the outstroke the film thickness in the textured zone is much greater than that in the non-textured zone, regardless of the texture shapes. It is reasonable because, as shown in Equations (3) and (7), the film thickness in the textured zone is *h*_r_ larger than that in the non-textured zone. In this case, the film thickness distribution is like the shapes of the rod’s surface micro-cavity. Since the film thickness in the textured zone is much bigger than that in the non-textured zone, less asperity contact will exist in the textured zone, resulting in weaker seal wear.

Figure 12 shows the film thickness distributions during the instroke. As is the case during the outstroke, the film thickness in the textured zone is greater than that in the non-textured zone. Less asperity contact exists in the textured zone, and the seal wear is weaker. Comparing the film thickness during the outstroke and instroke, the film thickness during the outstroke is smaller than that during instroke, regardless of the smooth or textured rod. Hence, a stronger seal wear will occur during the outstroke.

**Figure 14:**
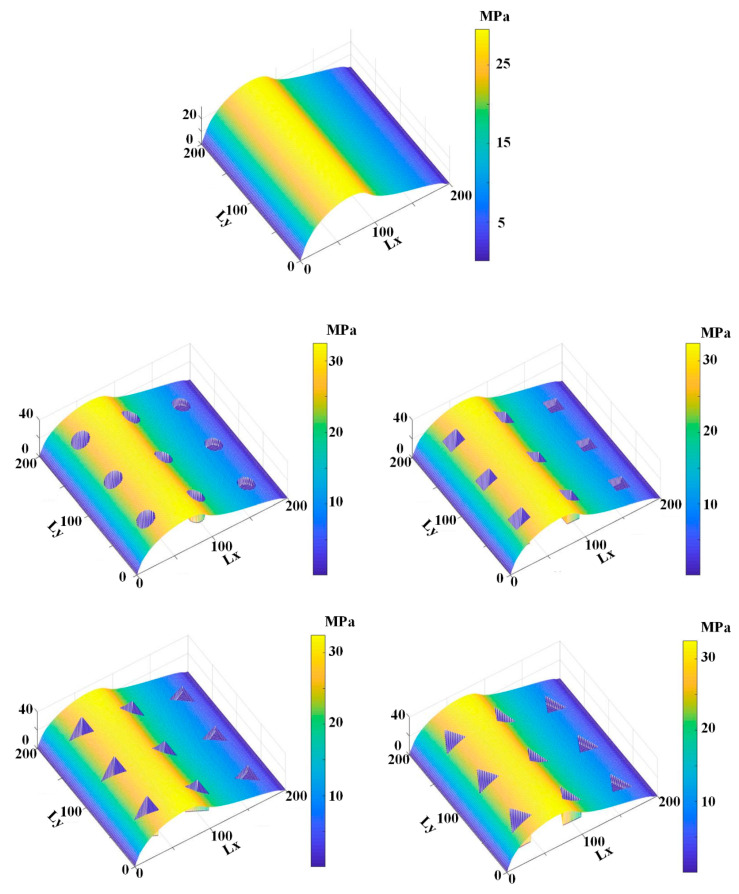
Asperity contact pressure during the instroke.

Figure 13 and Figure 14 show the asperity contact pressure distributions on the sealing zone with different textured rods. As shown in Figure 13, the asperity contact pressures of the textured rod are bigger than those of the smooth rod during the outstroke, except in the textured zone. As mentioned above, the film in the textured zone is very thick, resulting in less asperity contact existing. Seal wear in the textured zone is thus very weak. According to the micro deformation analysis of the seal surface, the pressure difference in the textured zone is undertaken by the fluid and asperity contact in the non-textured zone, the asperity contact pressure in the non-textured zone is thus bigger than that of the smooth rod. A stronger wear will exist on the seal surface in the non-textured zone.

Figure 14 shows the asperity contact pressure distributions for the smooth and textured rods seal during the instroke. As is the case during the outstroke, the asperity contact pressures with the textured rods are bigger than those with the smooth rod. In the textured zone there is little existing asperity contact, resulting in a weaker seal wear. In the non-textured zone, since the asperity contact pressure is bigger than that of the smooth rod, a stronger seal wear will exist.

**Figure 15:**
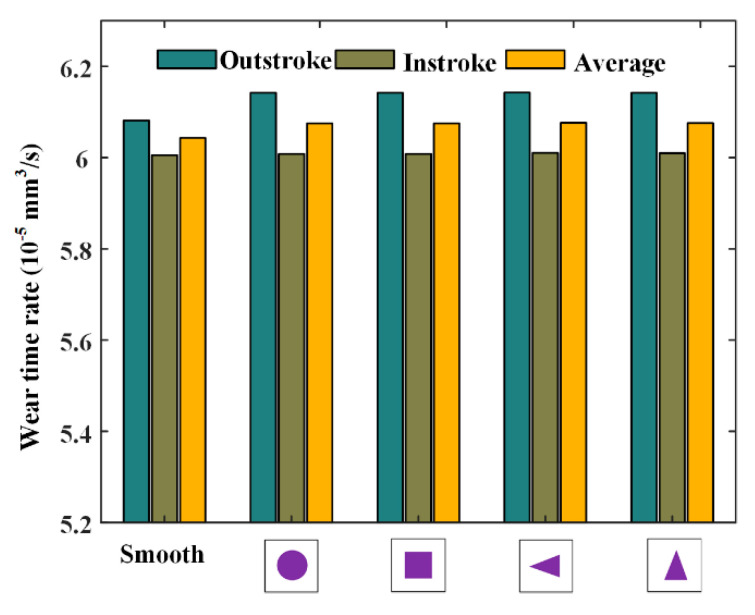
Wear time rates of different textures.

**Figure 16:**
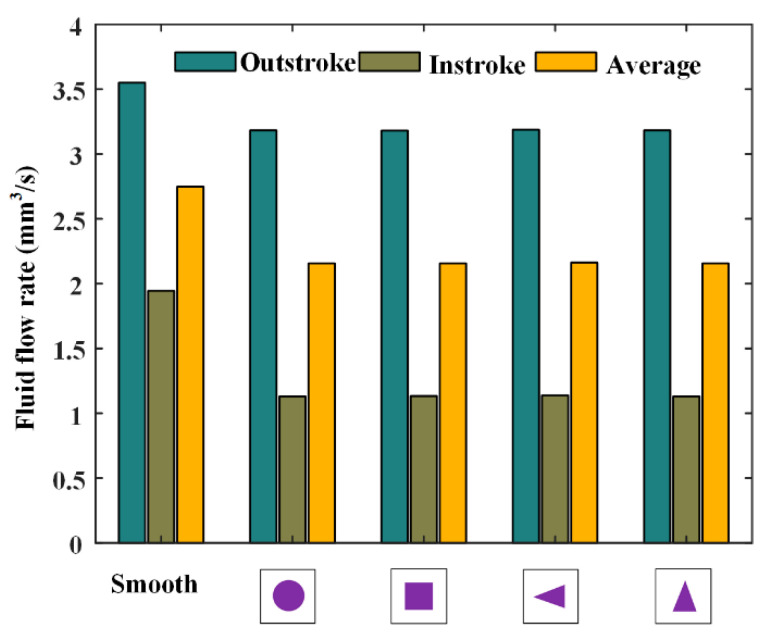
Fluid flow rates of different textures.

Figure 15 and Figure 16 show the simulation results of wear time rates and fluid flow rates, respectively. As shown in Figure 15, it can be noted that, during the outstroke, the textured rods will significantly increase the seal wear compared with the smooth rod, but during the instroke the increase is very small. The above-mentioned phenomena are in accordance with those in the analysis of the fluid pressure distribution. The average seal wear rate of the outstroke and instroke with the textured rod is a little bigger than that with smooth rod. Hence, it can be concluded that the effect of the rod texture on the seal wear is negative. In addition, it can be noted that the effects of the textures on the seal wear are almost identical.

Figure 16 shows the fluid flow rates during the outstroke and instroke for different kinds of textures. Note that the flow rate of the textured rod is a little bigger than that of the smooth rod, during both the outstroke and instroke. It should be emphasized that, since the rod speed is slow, the fluid is carried out of the cylinder during the instroke. Therefore, the seal will leak during the instroke. In this case, the textured rod can reduce the seal leakage during both the outstroke and instroke, as shown in Figure 16. Moreover, the effects of the textured rods on the seal leakage are basically the same.

**Figure 17:**
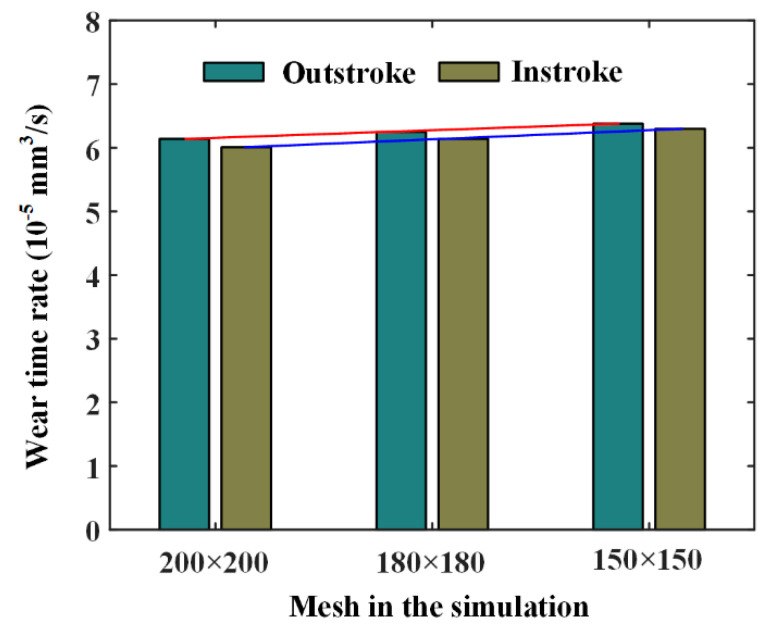
Wear rates under different simulation meshes.

In the numerical calculation of the seal wear, simulation space on the sealing zone should be meshed. When the mesh is 200 × 200, 180 × 180, and 150 × 150, the simulation results of the circle texture are shown in Figure 17. It can be seen that the simulation wear rate is affected by the calculation mesh. When the mesh changes from 200 × 200 to 150 × 150, the value of the wear rate increases by 3.9% during outstroke, and increases by 4.8% during instroke. To this end, we need to increase the element density of the mesh in the numerical simulation to improve the accuracy of the wear calculation, although the simulation time is thus increased.

### 6.3. Effects of Rod Speed

**Figure 18:**
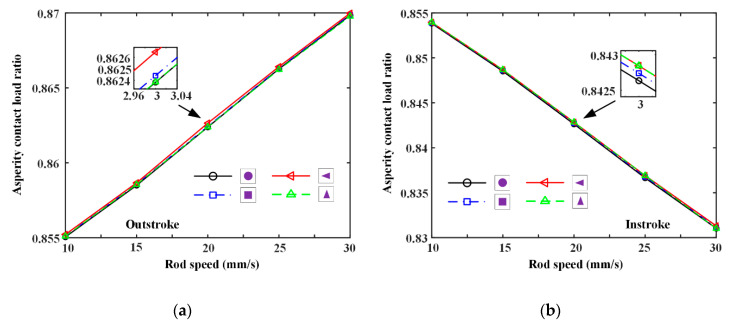
The relationship between the rod speeds and asperity contact load ratios, (**a**) outstroke, (**b**) instroke.

**Figure 19:**
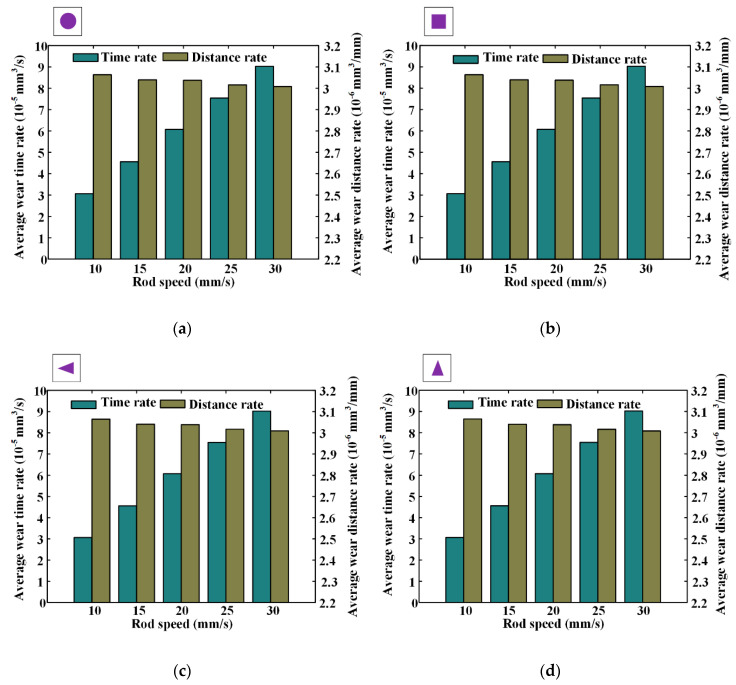
The wear rates under different rod speeds, (**a**) circle, (**b**) square, (**c**) triangle (Axial symmetry), (**d**) triangle (Circumferential symmetry).

Seal wear with different textured rods under different rod speeds is also simulated, as shown in Figure 18 and Figure 19. In Figure 18, the asperity contact load ratio increases with rod speed increasing during the outstroke, regardless of the texture shapes. However, during the instroke the contact load ratio increases. It indicates that, during the instroke the fluid pressure increases with rod speed, and the seal lip is thus pushed higher, moving away from the rod surface. However, during the outstroke the fluid pressure is weakened by rod speed increasing, resulting in the rod’s surface being closer to the lip seal. In addition, the asperity contact load ratios of different textures are similar to each other, which is in accordance with the analysis of Figure 13 and Figure 14. Hence, the seal wear of different textured rods is similar to each other too. Through Figure 18, it can be seen that the operating conditions would significantly affect the lubrication of the rod seal, seal wear is thus changed with the operating conditions. Asperity contact load ratios during the outstroke are a little bigger than those during the instroke. Hence, the wear of the reciprocating seal during the outstroke will be greater than that during instroke, regardless of the rod speed.

The average wear rate is defined as the mean value of the outstroke and instroke, and the simulation results of different textures are shown in Figure 19. Note that when the rod’s speed is increasing from 10 mm/s to 30 mm/s, the average wear distance rates decrease from about 3.08 × 10^−6^ mm^3^/mm to about 3.02 × 10^−6^ mm^3^/mm. It is because of this, the increase in the seal wear during the outstroke is smaller than the seal wear decrease during the instroke. In addition, with the rod’s speed increasing, average wear time rates increase from about 3.1 × 10^−5^ mm^3^/s to about 9.1 × 10^−5^ mm^3^/s. It can be concluded that the service lifetime of the seal will be reduced with the increased rod speed. Therefore, when analyzing the seal wear with the textured rod, the operating condition of the rod’s speed should be considered.

### 6.4. Effects of Rod Texture

**Figure 20:**
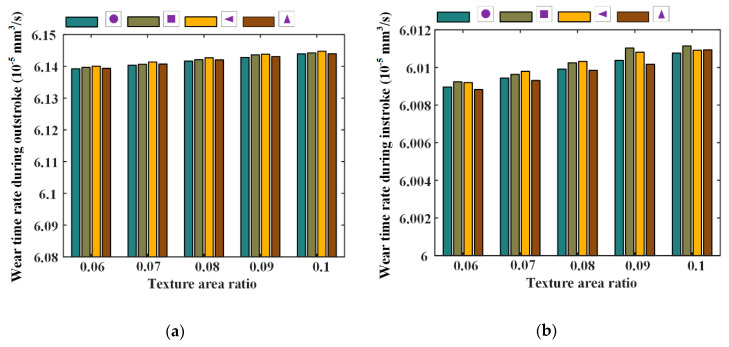
The relationship between wear time rate and texture area ratio, (**a**) outstroke, (**b**) instroke.

**Figure 21:**
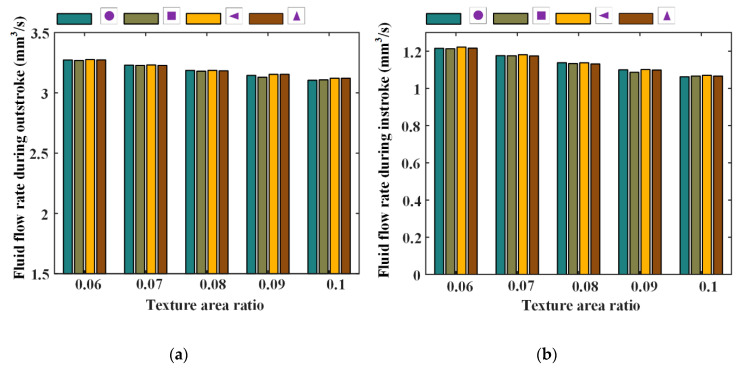
The relationship between wear time rate and texture area ratio, (**a**) outstroke, (**b**) instroke.

**Figure 22:**
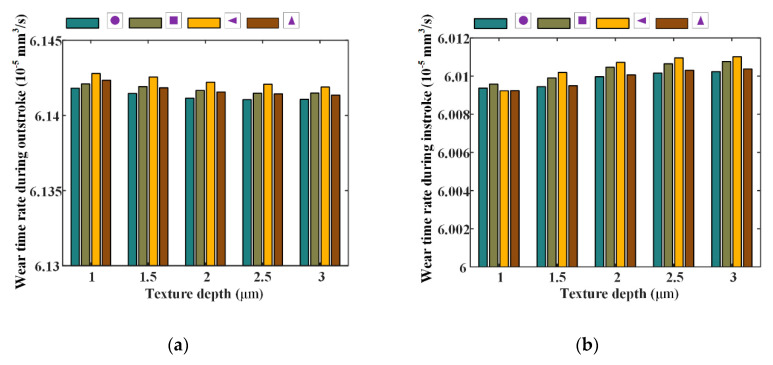
The relationship between wear time rate and texture depth, (**a**) outstroke, (**b**) instroke.

**Figure 23:**
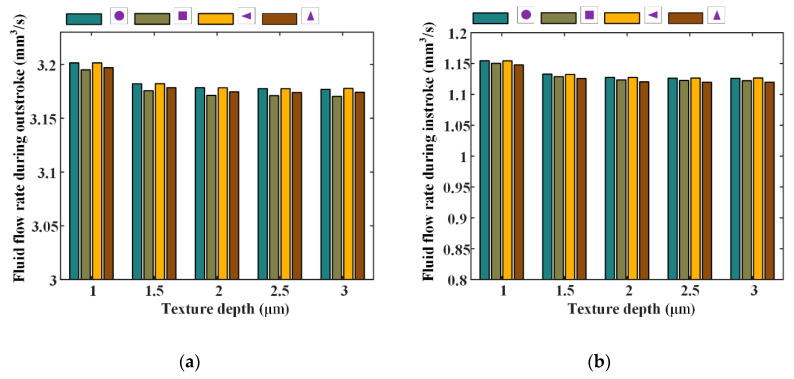
The relationship between wear time rate and texture depth, (**a**) outstroke, (**b**) instroke.

**Figure 24:**
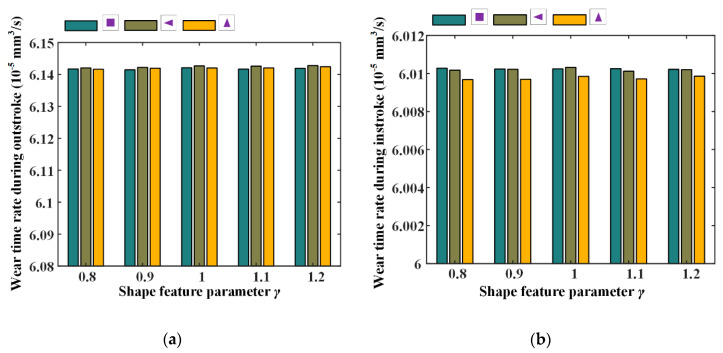
The relationship between wear time rate and texture shape parameter, (**a**) outstroke, (**b**) instroke.

**Figure 25:**
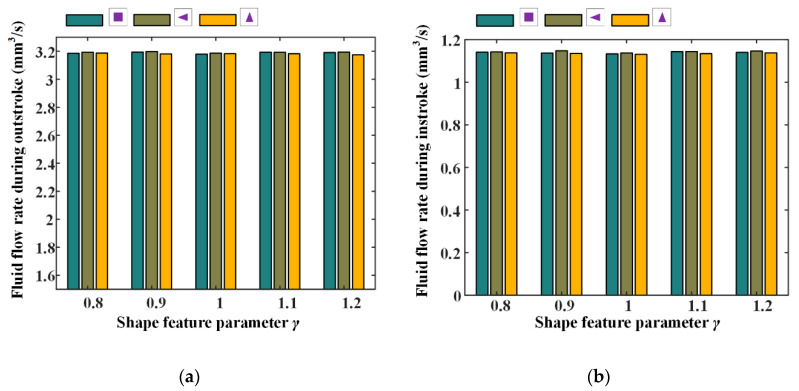
The relationship between fluid flow rate and texture shape parameter, (**a**) outstroke (**b**) instroke.

Under different texture parameters of the texture area ratio, depth, and ratio of the axial length of the micro-cavity to the circumferential length, seal wear and leakage of the reciprocating seal are simulated and the results are shown in Figure 20, Figure 21, Figure 22, Figure 23, Figure 24 and Figure 25.

Figure 20 and Figure 21 show the seal wear rates and fluid flow rates with different texture area ratios, respectively. Corresponding simulation parameters are: rod speed 20 mm/s, texture depth 1.3 μm, shape feature parameter one, and texture area ratio 0.06–0.1.

In Figure 20, it can be noted that, with texture area ratio increasing, wear time rates increase during outstroke, regardless of the texture shapes. During the instroke, the wear time rates increase too. It is because of this that with the texture area ratio increasing, the pressure difference in the textured zone increases, resulting in a bigger load on the non-textured zone. The sealing lip is thus closer to the rod’s surface and the lubricating film becomes thinner. In this case, the sear wear in the non-textured zone becomes worse. If the increase in the seal wear in the non-textured zone is bigger than the decrease in the textured zone, the seal wear will increase, as shown in Figure 20.

As shown in Figure 21, with the texture area ratio increasing, the fluid flow rates are decreased during both the outstroke and instroke. It is because that, with the increase of the texture area ratio, the pressure difference in the textured zone increases.The seal lip is thus pushed closer to the rod’s surface and the film thickness in the sealing zone becomes thinner. Hence, the fluid flow rate of the seal is decreased with the increasing texture area ratio.

Figure 22 and Figure 23 show the seal wear rates and fluid flow rates with different texture depths, respectively. The corresponding simulation parameters are: rod speed 20 mm/s, texture area ratio 0.08, shape feature parameter one, and texture depth 1–3 μm.

As shown in Figure 22, it can be noted that, with texture depth increasing the wear of the seal decreases during the outstroke, regardless of the texture shapes. However, during the instroke the seal wear increases when the texture depth increases. This means that, with texture depth increasing, the fluid pressure increases during the outstroke, while it decreases during the instroke. As shown in Figure 23, it can be noted that the fluid flow rates decrease with the increasing texture depth during the outstroke, regardless of the texture shapes. This is because that, although the fluid pressure in the textured zone increases during the outstroke, when the texture depth increases, the asperity contact pressure in the textured zone decreases, the whole pressure in the textured decreases. In this case, the asperity contact pressure in the non-textured zone increases and the lubricating film on the sealing zone of the textured rod is decreased. Hence, the fluid flow rate decreases. During the instroke, the fluid flow rates decrease too. It is because of this that during the instroke the fluid pressure and the asperity contact pressure in the textured zone decrease with increasing texture depth. So, the asperity contact pressure in the non-textured zone increases. In this case, the lubricating film becomes thinner and the fluid flow rate decreases.

Through the analysis of Figure 20 and Figure 21, increasing the texture depth is a possible way to increase the seal performance, since it can reduce the leakage of the seal, while it may not increase the wear rate.

Wear time rates and fluid flow rates with different texture feature parameters were also numerically investigated, the simulation results are shown in Figure 24 and Figure 25. Corresponding simulation parameters are: rod speed 20 mm/s, texture area ratio 0.08, texture depth 1.3 μm, and shape feature parameter 0.8–1.2.

Figure 24 shows the wear time rates with different shape feature parameters, during the outstroke and instroke. Note that, with the feature parameter increasing, the wear time rates are nearly unchanged, regardless of the textures shapes. This is because with the feature parameter increasing, the pressure difference of the textured zone in the x-axis direction increases, and in the y-axis direction decreases, the pressure difference in the whole textured zone is thus unchanged. Hence, the load of the asperity contact on the sealing zone has no change, resulting in an unchanged of the seal wear rate.

The fluid flow rates with different shape feature parameters of the textures are shown in Figure 25. It can be seen that the fluid flow rates have no change with the increasing feature parameter. As mentioned above, with the shape feature parameter increasing, the pressure difference in the whole textured zone is unchanged, resulting in an unchanged load in the non-textured zone. So, the film thickness on the sealing zone is unchanged, and the fluid flow rate has no change.

## 7. Conclusions

In this paper, a numerical model is presented to investigate the effects of the textured rods on the wear of the reciprocating seal. This model is focused on the seal wear under mixed lubrication conditions by combining the EHD lubrication model and the Archard wear model. An iterative algorithm is used to solve the lubrication model, since the fluid pressure, asperity contact pressure and micro-deformation of the seal lip are strongly coupled. A comparison of the average asperity contact pressure on the simulation space between the present model and Huang’s model is carried out to validate the model in the present research. The effects of the mesh in the numerical simulation are analyzed. Seal wear under different rod speeds are simulated and analyzed. Importantly, the effects of the texture on the seal wear are parametrically studied.

The textures on rod surface have a significant influence on the fluid pressure, film thickness and asperity contact pressure distributions. Since the wear of the seal lip greatly depends on the asperity contact of the seal with the rod surface, the texture effects cannot be neglected. Compared with the smooth rod, the textured rod will increase the seal wear, but reduce the seal leakage. Under the same sealed pressure, the seal during the outstroke seems to have a higher risk of wear than during the instroke, and should be receive more attention in the seal design. With the texture area ratio increasing, the seal wear increases and the leakage decreases, regardless of the texture shapes. When the texture depth increases, the seal leakage decreases, the seal wear decreases during the outstroke and increases during the instroke. The shape feature parameter has no effect on the wear and the leakage of the reciprocating rod seal. In addition, rod speed is one of the most important factors for analyzing the seal wear and needs to be considered in the analysis of the seal wear with textured rods. The simulation results are sensitized to the mesh in that it needs to increase elements density of the mesh to improve the accuracy of the wear calculation.

The presented research provides a foundation for engineers to investigate the seal wear of the reciprocating seal with textured rods. The effects of start/stop of the rod are ignored in the present model, but the start/stop of the rod exacerbates the seal wear since the seal surface traps in the micro-cavity on rod surface when the rod is stationary. Therefore, the effects of start/stop of the rod and experimental verifications should be focused on in future research.

## Figures and Tables

**Table 1:** Parameters of the step seal.

Parameter	Value
*C*_10_	1.87 MPa
*C*_01_	0.47 MPa
*d*	0.000113
$E_{seal}$	250 MPa
$\upsilon_{seal}$	0.4

**Table 2:** Main parameters in the simulations.

Parameter	Meaning	Value	Unit
*σ*	Root mean square roughness	0.8	μm
*D*_rod_	Diameter of the rod	25.4	mm
*p*_s_	Sealed pressure	5	MPa
*p*_a_	Ambient pressure	0.1	MPa
*R*	Asperity radius	0.8	μm
*η*	Asperity density	1 × 10^5^	mm^−2^
*μ*_0_	Fluid viscosity	0.0387	Pa·s
*k*	Wear modulus	1.2 × 10^−5^	mm^3^/Nm
*u*	Rod speed	10–30	mm/s

**Table 3:** Values of the rod texture parameters.

Number	Texture	Depth	Area Ratio	Shape Feature Parameter
a	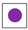	1.3 μm	0.08	-
b	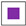	1.3 μm	0.08	1
c	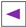	1.3 μm	0.08	1
d	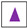	1.3 μm	0.08	1

## List of Symbols

*a* length of micro-cavity in axial direction	*b* length of micro-cavity in circumferential direction
*C*_10_, *C*_01_, *d* Mooney–Rivlin coefficients	*D*_rod_ rod diameter
*E* equivalent Young’s modulus	*E*_seal_ Young’s modulus of the seal
*F* cavitation factor	*F*_sum_ sum of the pressure differences
*h* film thickness	*h*_0_ initial film thickness
*h*_p_ depth of the micro-cavity	*h*_r_ rod surface height
*h*_T_ average truncated film thickness	*h*_w_ wear depth
Δ*h* micro deformation of the sealing lip surface	*H* hardness
*I*_1_, *I*_2_ deviatoric strain invariants	*J* parameter relating to the elastic deformation gradient
*k* wear modulus	*K* wear coefficient
*L* side length of texture	*L*_x_ length of the simulation space in axial direction
*L*_y_ length of the simulation space in circumferential direction	*p* static contact pressure
*p*_a_ ambient pressure	*p*_c_ asperity contact pressure
*p*_cav_ cavitation pressure	*p*_f_ fluid pressure
*p*_n_ normal contact pressure	*p*_s_ sealed pressure
Δ*p* pressure difference	${\bar{p}}_{c}$ averge asperity contact pressure
*r*_c_ radius of circular micro-cavity	*r*_s_ wear distance rate
*r*_t_ wear time rate	*R* asperity radius of the seal
*S* relative sliding distance	*u* rod speed
*V* material wear volume	*W* normal load
$\tilde{W}$ strain energy density	*x* coordinate in x-axis direction
*y* coordinate in y-axis direction	*η* asperity density of the seal
*γ* ratio of the axial length of micro-cavity to the circumferential length	*μ* fluid viscosity
*μ*_0_ fluid viscosity under the ambient pressure	*σ* seal surface roughness
*σ*_s_ equivalent standard deviation of surface roughness	*ϕ*_x_, *ϕ*_y_ pressure flow factors
*ϕ*_s.c.x_ shear flow factor	*Φ* fluid pressure or density of the cavitation region
*υ*_seal_ Poisson’s ratio of the seal	
